# Performance of an Innovative Low-Cost Recycled Filling (LCRF) in Anaerobic Treatment of Dairy Effluent—A Pilot-Scale Study

**DOI:** 10.3390/ma15217815

**Published:** 2022-11-05

**Authors:** Marcin Zieliński, Marcin Dębowski, Joanna Kazimierowicz

**Affiliations:** 1 Department of Environmental Engineering, Faculty of Geoengineering, University of Warmia and Mazury in Olsztyn, 10-720 Olsztyn, Poland; 2 Department of Water Supply and Sewage Systems, Faculty of Civil Engineering and Environmental Sciences, Bialystok University of Technology, 15-351 Bialystok, Poland

**Keywords:** dairy effluent, anaerobic reactor, active filling, anaerobic digestion, phosphorus removal, biogas, bacterial community

## Abstract

The rapid growth in dairy production leads to increasing outputs of high-load effluent, necessitating new methods of treating such waste. Anaerobic processes have been increasingly popular but are hamstrung by limited nutrient removal efficiency. The aim of the present study was to investigate whether low-cost recycled filling (LCRF) improves the anaerobic treatment of dairy effluent. The addition of LCRF was found to increase both COD removal (86.1 ± 2.6%–92.8 ± 1.6%) and P_tot._ removal (22.1 ± 3.5% to 36.9 ± 4.6%) from the wastewater. The LCRF ensured near-neutral pH and stabilized the structure of the anaerobic microbe community (including Archaea) across all pollutant loads tested. This translated to efficient biogas production and high methane content in the LCRF reactors, peaking at 0.35 ± 0.01 m^3^/kg COD_removed_ and 68.2 ± 0.6% (respectively) in the best-performing variant.

## 1. Introduction

According to the Food and Agriculture Organization of the United Nations (FAO) data from 2020, the global market for milk is approximately 852 million tons and is steadily growing [1,2]. The largest producers are India, the European Union (EU) and the USA, at 196.2 million, 167.4 million and 99.2 million tons produced, respectively. The Organization for Economic Co-operation and Development (OECD) expects global annual milk production to reach 997 million tons by 2029 [3,4]. This growth in milk production goes hand in hand with the increased generation of wastewater, whose properties make it dangerous to aquatic ecosystems [5,6]. According to the European Dairy Association (EDA), liquid waste from dairy production mostly consists of waste milk and process waste (including industrial cleaning and rinsing water), with small volumes of sanitary wastewater [7,8]. Milk and fat losses during cheese production are estimated at 0.2% and 0.1% (respectively), whereas during drinking milk production, are 1.9% and 0.7%, respectively [8]. Dairy wastewater contains large amounts of organic and biogenic compounds, though actual levels observed have proven highly variable due to differences in water volumes used, types of dairy produced, and processing methods [9]. Water input needed to produce 1 m^3^ of processed milk can be anywhere from 1 to 10 m^3^, leading to differences in wastewater output and pollutant concentrations [10].

The high pollutant levels in dairy wastewater can aggravate eutrophication and degradation of natural water bodies if discharged directly into the environment [11]. Therefore, effective methods are needed to remove pollutants and prevent negative impacts on receiving water bodies [12]. Anaerobic wastewater treatment (AWT) is one such process, which has been growing in popularity [13]. The prevalence of anaerobic digesters stems from their many major advantages over aerobic activated-sludge or biofilter processes. [14]. The most frequently cited benefits are: effective treatment of high-OLR sewage, ability to accommodate high OLRs, relatively small size and space requirements as well as markedly lower investment and operation costs compared with aerobic technologies [15]. Anaerobic systems generate less surplus sludge, provide better sludge stabilization and lead to improved sanitary indicators, which directly facilitates neutralization and management. A key feature is their ability to produce methane-rich biogas, which can be harnessed for energetic purposes [16].

The low nitrogen and phosphorus removal rates of AWT are considered to be its biggest drawbacks [17]. These biogenic substances are the main factors responsible for the rapid eutrophication and degradation of natural water bodies [18]. Fixation of nitrogen and phosphorus in digesters is driven solely by the growth of bacterial biomass, of which they are important building blocks. The N and P removal percentage is usually within single digits [19]. The resultant anaerobically treated effluent cannot be discharged directly to a receiving water body, as it exceeds permissible concentrations of N and P. Therefore, there is a real need to seek new and improved processes for better organic compound removal, nutrient take-up and biogas production.

The aim of the present study was to test waste-derived, low-cost filling in the treatment of real-world dairy wastewater in pilot-scale anaerobic reactors as well as to establish how different digester loadings affect organic compound removal, nutrient take-up, biogas composition and yield as well as the structure of the bacterial taxa. Anaerobic digestion (AD) performance was compared against lightweight expanded clay aggregate filling (LECAF) reactors.

## 2. Materials and Methods

### 2.1. Location

LCRF performance was tested on real-world dairy effluent in pilot-scale anaerobic reactors located in the Zakład Mleczarski Sp. z o.o. dairy production facility in Łaszczów, Poland (N 50°31′35.915″, E 23°42′58.623″). The factory produces skimmed-milk powder, sweetened condensed milk, whey powder, ripened Dutch cheeses, mozzarella cheeses, cagliata cheeses and butter. 

### 2.2. Experimental Design

The experiment was divided into 2 series with different fillings (packing) used in the anaerobic reactors. Lightweight expanded clay aggregate filling (LECAF) was used in series 1, whereas low-cost recycled filling (LCRF) was tested in series 2. The pilot-scale experiment was run for 10 months. For the first 2 months, the microbes gradually adapted to the medium, and bacterial microflora was allowed to build up on the surface of the tested fillings. Then, the exact experiment was performed for the subsequent 8 months, designed to assess the wastewater treatment performance and biogas production capacity. Each experimental series included 5 process variants (V) with different organic load rates (OLRs) in the digesters. OLR was changed every two months: V1–3.0 kg COD/m^3^·d (months 0–2, adaptation), V2–4.0 kg COD/m^3^·d (months 3–4), V3–5.0 kg COD/m^3^·d (months 5–6), V4–6.0 kg COD/m^3^·d (months 7–8), and V5–7.0 kg COD/m^3^·d (months 8–10). Hydraulic retention time (HRT) was systematically reduced to achieve the progressively higher OLRs: V1–48 h, V2–36 h, V3–29 h, V4–24 h, and V5–20 h.

### 2.3. Materials

#### 2.3.1. Dairy Wastewater

The dairy wastewater used for the experiment was sourced from the retention tank of the on-site wastewater treatment system at the dairy factory (HRT = 24 h). A pumping system conveyed the effluent to the experimental pilot-scale bioreactors. The profile of the raw dairy wastewater is given in Table 1.

#### 2.3.2. Anaerobic Sludge

Anaerobic sludge (AS) was sourced from an enclosed digester chamber in which activated sludge from an aerobic treatment system (dairy-factory oxidation ditch) was anaerobically digested. The process parameters for the digester were: OLR = 2.5 kg VS/m^3^·d, HRT = 20 d and temperature = 37 °C. AS levels in the digester were maintained at an average 4000 ± 500 g dry mass/m^3^. The fillings (LECAF, LCRF) were inoculated with AS over a period of 20 weeks, as the experimental system was fed with the dairy effluent + sludge mixture. AS concentration in the wastewater was kept at approximately 2000 ± 400 g dry mass/m^3^. The AS profile is given in Table 2.

#### 2.3.3. Lightweight Expanded Clay Aggregate Filling (LECAF)

**Figure 1:**
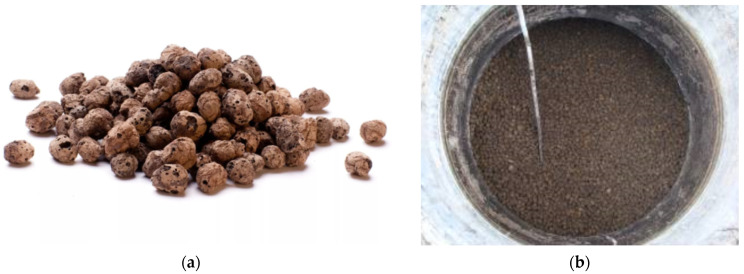
Light expanded clay aggregate used as anaerobic reactor filling in the experimental variant 1 ((**a**) general view of the grains; (**b**) LECA in the digester).

In variant 1, the anaerobic reactors were packed with 10-20R light expanded clay aggregate (LECA^®^ Sp. z o.o., Gniew, Poland). LECA is a porous, lightweight and tough ceramic aggregate made by heating and expanding loamy clay in rotary kilns at 1150 °C (Figure 1). Key parameters of the filling were as follows: EN 15732 and EN 13055-1 compliant, particle size: 8–20 mm, shape: round, bulk density (loose): 246–333 kg/m^3^ (averaging approximately 290 kg/m^3^), minimum crushing strength: 0.75 N/mm^2^, tensile strength > 500 kPa, heat transfer coefficient: 0.095–0160 W/mK, critical angle of repose: 45°, absorption capacity < 35%, CE 04/EN 13055-1/0770-CPR-2370-05-17, freeze resistance < 0.8%. The light aggregate behaves similarly to a fluid. The reactors were packed with 3.0 m^3^ of LECA.

#### 2.3.4. Low-Cost Recycled Filling (LCRF)

**Figure 2:**
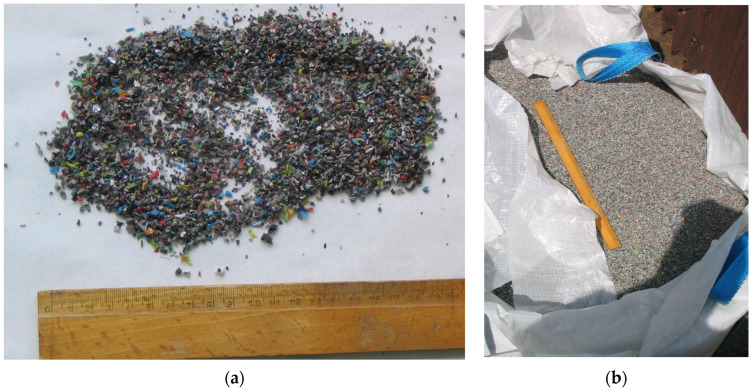
Waste material used to produce the LCF ((**a**) particle size and structure; (**b**) transport and storage container).

**Figure 3:**
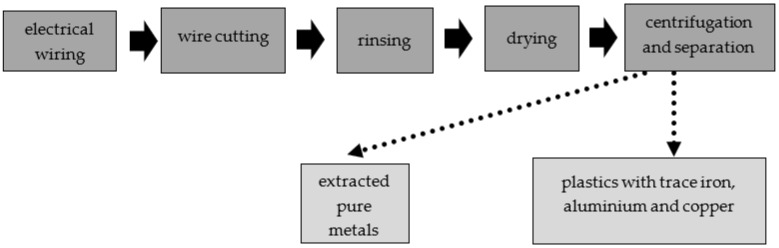
Step-by-step preparation of material for the active filling.

The LCRF was made from commercially available waste (leftover mixed plastics from metal recycling of scrap wiring and electrical systems, mostly junked cars). The waste plastics consisted of various insulating materials (normal rubber, silicone rubber, halogen-free material, cross-linked polyethylene, organohalogen material, softened PVC coating and heat-resistant softened PVC coating), as well as some iron, copper and aluminum. The metal accounted for approximately 10% of the material by weight, including approximately 5 %_w/w_ aluminum, 3%_w/w_ iron and 2%_w/w_ copper (Figure 2). To obtain the material for the LCRF, the electrical wires were first shredded. The disintegrated waste was then rinsed (to purge it of any contaminants) and dried. The resultant intermediate was passed through a system of centrifuges and sieves to separate and filter out waste plastics containing trace iron, copper and aluminum. The step-by-step preparation of the LCRF material is presented in Figure 3.

**Figure 4:**
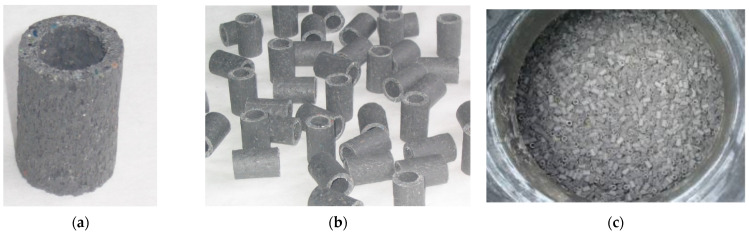
LCRF elements ((**a**) a single LCRF piece with visible porosity; (**b**) extruded filling; (**c**) LCRF in the anaerobic reactor).

The waste was processed in a mixer mill (RG-1820, Wartacz P.H.U., Wroclaw, Poland), to ensure consistent structure and quality of the final product. The substrate mixture was melted at a temperature of 110 °C to 160 °C into a uniform plastic mass. This mass was then fed through a single-screw extruder with a closed-tube pressing module (T-32-25, ZMC Metalchem Sp. z o.o., Cracow, Poland). The processed material was cut with a circular knife (N-100, Zamak Mercator Sp. z o.o., Skawina, Poland) to a length of 24 mm. Finally, the pieces were placed in a cooling trough (18 ± 2 °C) to bring their temperature down. LCRF elements are presented in Figure 4. The anaerobic reactors were filled with 3.0 m^3^ LCRF. The technical parameters of the LCRF structures are as follows: height: 24 mm, inner diameter: 10 mm, outer diameter: 18 mm, bulk density: 300 kg/m^3^, active surface area: 500 m^2^/m^3^, lengthwise crush strength: 0.04 N/mm^2^, widthwise crush strength: 3.2 N/mm^2^ and volatile matter content: 65%.

### 2.4. Pilot-Scale Anaerobic Reactor Station

**Figure 5:**
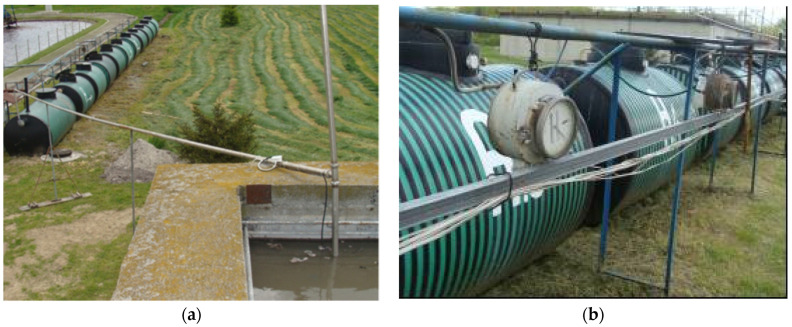
Anaerobic Reactor Station ((**a**) retention basin with influent pumping system; (**b**) anaerobic reactors).

Raw dairy effluent was conveyed into a retention basin with an active volume of V = 5000 m^3^. From there, the wastewater was pumped via ø 50 mm hoses into the experimental anaerobic reactor system. A single anaerobic reactor had an active volume of V = 3.0 m^3^ (in addition to the space taken up by the filling). The entire system was made up of six anaerobic tanks. Three were packed with LECAF (series 1) and the other three with LCRF (series 2). The wastewater feed was placed at the bottom of the anaerobic reactors to maintain an upflow. The temperature in the pilot-scale station was kept at 37 °C. The retention tank and the station’s raw-wastewater pumping system are presented in Figure 5a. The experimental anaerobic reactors are presented in Figure 5b.

### 2.5. Analytical Methods

The chemical oxygen demand (COD), total nitrogen (TN) and total phosphorus (TP) in the wastewater and effluent were analyzed once every 24 h using a spectrophotometer (Hach DR 6000, Düsseldorf, Germany). The content of total solids (TS) was determined gravimetrically. The pH was measured with a pH meter (1000 L, VWR International, Radnor, PA, USA). Biogas flow rate was measured continuously using a digital gas flow meter (Aalborg Instruments & Controls, Inc., Orangeburg, NY, USA). Biogas composition was analyzed once every 7 days using a GMF 430 m (GasData, UK) and a gas chromatograph (GC, 7890A Agilent, Santa Clara, CA, USA) equipped with a thermal conductivity detector (TCD). The GC was fitted with two Hayesep Q columns (80/100 mesh), two molecular sieve columns (60/80 mesh) and a Porapak Q column (80/100) operating at a temperature of 70 °C. The temperature at the injection and detector ports was 150 °C and 250 °C, respectively. Helium and argon were used as the carrier gases at a flow rate of 15 mL/min. Samples for molecular profiling of the bacterial community were extracted from filling elements at the end of each variant. The molecular analysis was performed to determine the percentage of ammonia-oxidizing bacteria (AOB) in the biofilm using the fluorescent in situ hybridization (FISH) technique [20].

### 2.6. Statistical Analysis

The statistical analysis was conducted using STATISTICA 13.3 PL. One-way analysis of variance (ANOVA) was used to determine significant differences across the groups. Significant differences between the variables were determined with Tukey’s HSD (*p* = 0.05). The experimental variants and analyses were carried out in triplicate.

## 3. Results and Discussion

### 3.1. Organic Matter Removal

**Figure 6:**
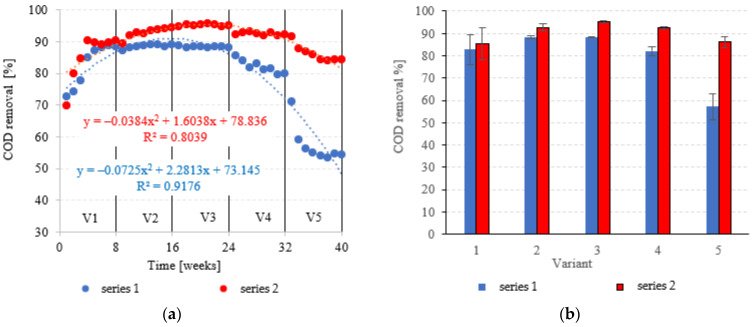
COD removal ((**a**) changes throughout the experiment; (**b**) average across experimental variants).

**Figure 7:**
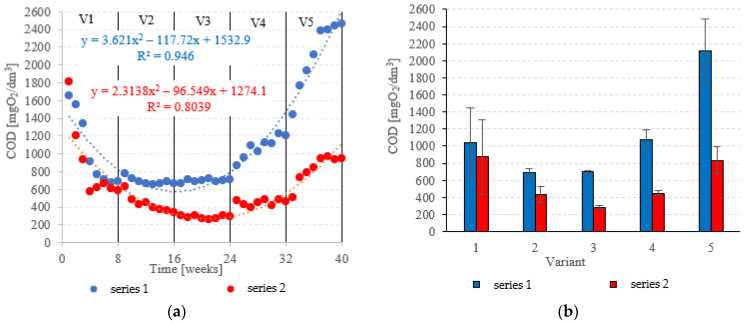
COD in the treated effluent ((**a**) throughout the experiment; (**b**) average across experimental variants).

COD in the influent was 6020 ± 595 mgO_2_/dm^3^. COD removal in series 1 (LECAF) reached 82.8 ± 6.8% during the adaptation period (V1). In V1–V2, biodegradation efficiency ranged from 88.5 ± 0.7% to 88.4 ± 0.3% (Figure 6a,b), resulting in COD levels in the final effluent of approximately 700 mgO_2_/dm^3^ (Figure 7a,b). V4 suffered a significant drop in removal rates (82.1 ± 2.0%), but a much more dramatic decline (Figure 6a,b) occurred after increasing the OLR to 7.0 kg COD/m^3^·d (V5) when COD removal dropped to 57.2 ± 5.9%, leading to an increase in COD in the treated effluent to a level of 2120 ± 370 mgO_2_/dm^3^ (Figure 7a,b). The LCRF used in series 2 led to a significantly better and more consistent wastewater treatment performance within the tested OLR range of 4.0 kg COD/m^3^·d to 7.0 kg COD/m^3^·d. COD removal ranged from 95.2 ± 0.3% in V3 to 86.1 ± 2.6% in V5 (Figure 6a,b). COD in the final effluent was below 900 mgO_2_/dm^3^ across all LCRF variants (Figure 7a,b). Many different anaerobic reactor designs are used to treat dairy wastewater [21]. The literature data indicate that organic removal rates as high as 98% can be achieved at loads of up to 9.8 kg COD/m^3^·d, provided a very long HRT is maintained (142 days) [22]. Researchers have also considered anaerobic filter and fluidized beds [23,24,25,26]. Purushothaman et al. [25] have used a fluidized-bed reactor to biodegrade dairy effluent on a wood-particle medium, removing 84% COD at 30 °C and pH 7. Kundu et al. [27] treated simulated dairy effluent in a fluidized-bed anaerobic reactor, achieving a COD of 78% at an OLR of 8 kg COD/m^3^·d. Dębowski et al. [28] employed an innovative multi-section horizontal flow anaerobic reactor (HFAR) to treat dairy effluent, removing approximately 85% COD at 1.0–2.0 kg COD/m^3^·d OLR. Others [23] have shown that inverse fluidization could also be employed in AD of dairy effluent, achieving a 90% COD removal rate with an OLR of 10 kg COD/m^3^·d.

**Figure 8:**
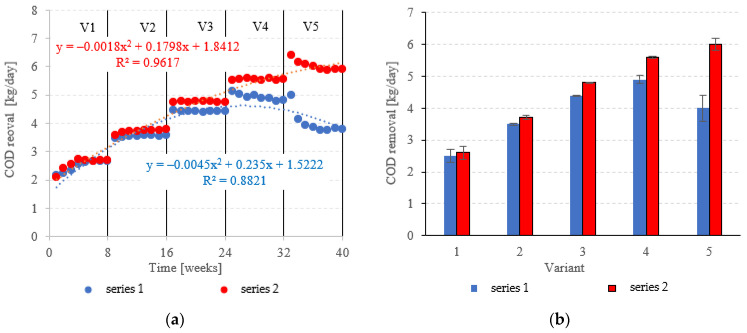
COD removed ((**a**) throughout the experiment; (**b**) average across experimental variants).

COD removed in the LECAF series ranged from 2.5 ± 0.2 kg COD/day (adaptation period) to 4.9 ± 0.12 kg COD/day at 6.0 kg COD/m^3^·d OLR. In the LCRF series, the removal rate was 2.6 ± 0.22 kg COD/day in V1 to 6.0 ± 0.18 kg COD/day in V5 (Figure 8a,b). LCRF was also shown to maintain high treatment performance in the upper range of OLRs. A significant, but relatively small decline in COD removal was noted for 7.0 kg COD/m^3^·d OLR (Figure 8a,b). In contrast, the biodegradation of organics in the LECAF series was significantly impaired as early as V4, and increasing the OLR to 7.0 kg COD/m^3^·d led to an even sharper drop in performance (Figure 8a,b). Studies to date have estimated COD removal in packed anaerobic reactors at between 65% and 97.5% [29,30,31]. Najafpour et al. [32] investigated trends in organics removal from dairy effluent across an OLR range of 7.9 to 45.42 kg COD/m^3^·d. The best COD removal rates (97.5% and 88%) were recorded for 48 and 36 h HRT, respectively. The process performed substantially worse at 25 kg COD/m^3^·d OLR. A study on a magneto-active hybrid anaerobic biofilm reactor produced the highest COD removal rates (80%) at the OLR range of 6.0–8.0 kg COD/m^3^·d [33]. In an anaerobic baffled and biofilm reactor with magneto-active filling, COD removal was found to vary between 77% and 86% at an OLR of 10 kg COD/m^3^·d [34]. In other types of reactors, high COD removal rates of over 1000 kg COD/d were observed within the OLR range of 15 to 25 kg COD/m^3^·d [35].

### 3.2. Biogenic Pollutant Removal

**Figure 9:**
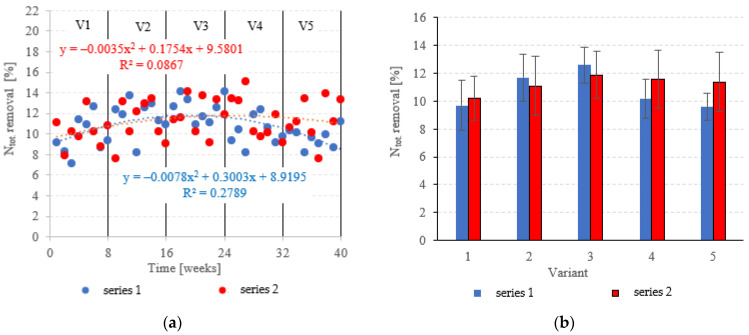
N_tot._ removal ((**a**) changes throughout the experiment; (**b**) average across experimental variants).

**Figure 10:**
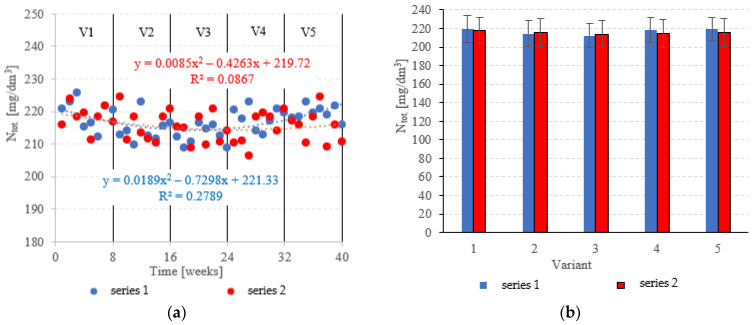
N_tot._ in the treated effluent ((**a**) throughout the experiment; (**b**) average across experimental variants).

Neither the choice of filling nor the OLR was found to significantly correlate with N_tot._ removal from dairy effluent (Figure 9a,b). In series 1 (LECAF), N_tot_ removal ranged from 9.6 ± 1.0% in V5 to 12.6 ± 1.3% in V3 (Figure 9a,b), with final concentrations of 219.6 ± 2.3 mgN_tot._/dm^3^ to 212.5 ± 3.0 mgN_tot._/dm^3^ (Figure 10a,b). Series 2 (LCRF) produced similar results–N_tot._ removal fell within the narrow range of 10.2 ± 1.6% (adaptation period–V1) to 11.9 ± 1.7% in the OLR = 5.0 kg COD/m^3^·d variant (Figure 9a,b). The final effluent contained between 214.0 ± 4.2 mgN_tot._/dm^3^ and 218.2 ± 3.8 mgN_tot._/dm^3^ (Figure 10a,b).

Anaerobic nitrogen removal has been shown to be an inefficient process fueled exclusively by microbial biomass growth [36]. In AD reactors, organic N species can only be converted into ammoniacal nitrogen [37], so further processing is necessary to remove nitrogen completely from the wastewater [38]. It has been posited that improving N removal in anaerobic wastewater treatment (AWT) systems is one of the biggest challenges for researchers [39,40]. Studies so far have looked at upgrading anaerobic processes with activated sludge [41], plant filters [42], zeolites [43], absorption methods [44], struvite production [45] or intensive microalgal cultivation systems [46].

**Figure 11:**
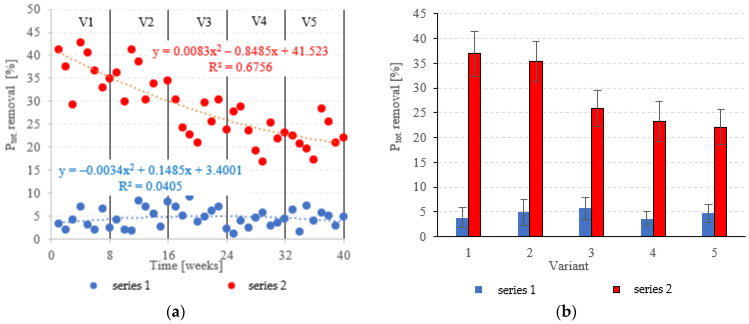
P_tot._ removal ((**a**) changes throughout the experiment; (**b**) average across experimental variants).

**Figure 12:**
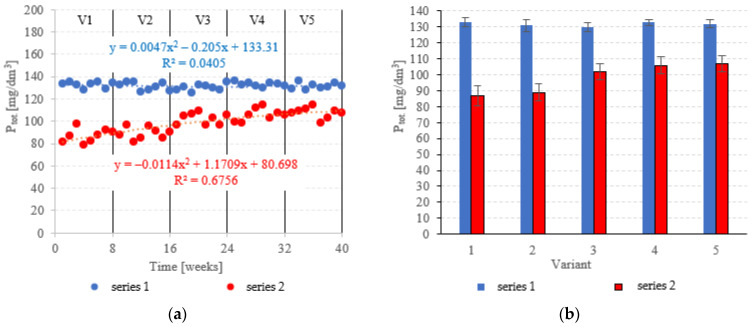
P_tot._ in the treated effluent ((**a**) throughout the experiment; (**b**) average across experimental variants).

The LECAF anaerobic reactors had little success in removing P_tot._, with removal rates falling within the narrow range of 3.6 ± 1.4% to 5.7 ± 2.2% (Figure 11a,b). The rates were statistically similar across all OLR variants. Concentrations of P_tot._ in the final effluent of series 1 (all variants) exceeded 130 mgP_tot._/dm^3^ (Figure 12a,b). The LCRF reactors performed much better in removing P_tot._. The removal efficiencies in V1 and V2 were 36.9 ± 4.6% and 35.4 ± 4.0%, respectively (Figure 11a,b), which translates to P_tot._ in the final effluent of less than 90.0 mgP_tot._/dm^3^ (Figure 12a,b). The higher OLRs of the subsequent variants had a significant negative effect on P_tot._ removal rates, which finally stabilized at 22.1 ± 3.5%–25.9 ± 3.7% (Figure 11a,b), with P levels in the final effluent at 102 ± 5.1 mgP_tot._/dm^3^ to 107 ± 4.8 mgP_tot._/dm^3^ (Figure 12a,b).

Mineralization is the only process by which N and P are removed under fermentative conditions [47]. The only way by which anaerobic bacteria can remove nitrogen and phosphorus is by absorbing them into their cells—a time-consuming process, as the microbes themselves are slow to grow [48]. Of course, converting organic P species into orthophosphates makes it easier to remove them in subsequent treatments [49]. Methods used to this end include an aerobic pass (activated sludge step) [50], biological beds, hydrophyte-based treatments, chemical precipitation with iron- or clay-based inorganic coagulants [51,52] and microalgae [46]. One approach increasingly discussed in the literature is the use of active fillings to promote phosphorus removal directly in the digester [53]. Many designs have been tested for dairy effluent treatment, including magneto-active hybrid anaerobic biofilm reactors (MA-HABRs) [33], anaerobic moving biofilm reactors (AMBRs) with iron-containing supports [54], anaerobic reactors with active filling (AF) heated with microwave radiation (EMR) [55] as well as anaerobic baffled and biofilm reactors with magneto-active packing media [34]. Removal performance has been reported to vary between 64.4% and 90.7%, depending on treatment parameters [29]. One of the reported drawbacks of active fillings for P removal is the issue of passivation–anaerobic biofilms forming a barrier around the packing media and reducing contact with the wastewater [56]. Consequently, the effectiveness of such packings often decreases the longer a reactor operates [19]. Another limitation is the depletion of the packing’s sorption capacity, after which it needs to be replaced or regenerated [29].

### 3.3. Evolution of pH and Bacterial Community

**Figure 13:**
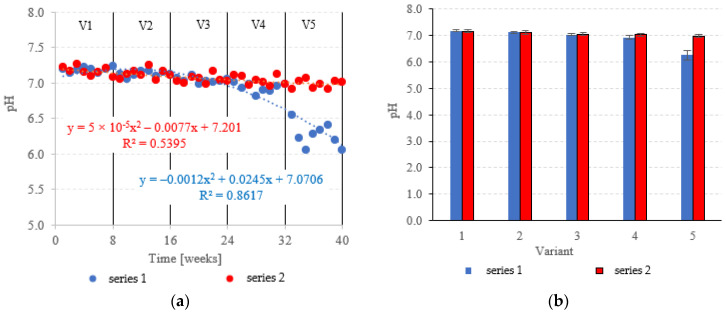
pH in the anaerobic reactors ((**a**) changes throughout the experiment; (**b**) average across experimental variants).

The pH was fairly stable across variants V1–V4 of the LECAF series, ranging from 6.93 ± 0.07 to 7.19 ± 0.03 (Figure 13a,b). Increasing the OLR to 7.0 kg COD/m^3^·d resulted in a sharp decrease in pH to 6.26 ± 0.17 (Figure 13a,b). LCRF reactors proved to be more resistant to OLR increases, with pH holding steady at a near-neutral level across all treatment variants. The pH fluctuated within the narrow range of 6.98 ± 0.06 in V5 to 7.17 ± 0.06 in V1 (Figure 13a,b). The stepwise increases of the OLR from 4.0 kg COD/m^3^·d to 7.0 kg COD/m^3^·d produced a consistent but non-significant decrease in average pH. The pH is a major determinant of AD performance [57]. A near-neutral pH in an anaerobic medium signifies that the hydrolysis, acidic and methanogenic phases of anaerobic conversion have reached an equilibrium [58]. It has been demonstrated that pH decreases are primarily driven by the accumulation of volatile fatty acids (VFAs), which are produced at the acidogenesis stage [59]. This usually happens at excessively high OLRs [60]. Decreased pH inhibits the activity of methanogenic bacteria, effectively reducing biogas production and pollutant removal rates [61]. This trend has been demonstrated by Zieliński et al. [35] in a study that examined dairy effluent treatment in a microwave radiation heated (MRH) multi-section hybrid anaerobic reactor (M-SHAR) and a conventional reactor. The reactor operated at steady state within the OLR range of 15 kg COD/m^3^·d to 20 kg COD/m^3^·d. Higher OLRs led to significant decreases in pH, negatively impacting digestion efficiency. Biogas yields dropped from 433 dm^3^/d (MRH reactor) and 384 dm^3^/d (conventionally heated reactor) to 324 dm^3^/d and 260 dm^3^/d, respectively [35]. Cheng et al. [62] have also shown a significant correlation between OLR and AD performance. OLR was found to have little adverse effect on the effluent quality and organic matter removal at levels up to 9.72 kg COD/m^3^·d. However, as the OLR was increased further to 14.58 kg COD/m^3^·d, the biogas production rate decreased significantly to 1.35 dm^3^/dm^3^·d [62]. Sanchez et al. [63] have also demonstrated that while higher OLRs can boost methane generation, they can also lead to system failure (due to the accumulation of VFAs and free ammonia) and decreased pH [63].

The anaerobic bacterial community was directly affected by the increasing OLRs, decreasing HRTs and the resultant changes in the reactor conditions, as shown in Table 3. The changing parameters had a particularly pronounced effect in the LECAF series. In variants V1–V3, the taxonomic structure consisted of 67 ± 11%–69 ± 9% Bacteria and 28 ± 5%–29 ± 7% Archaea. *Methanosarcinaceae* and *Methanosaeta* populations were also stable (14 ± 2%–15 ± 3% and 9 ± 2%–9 ± 3%, respectively). As the OLR reached 6.0 kg COD/m^3^·d, the taxonomic structure of anaerobic bacteria started to experience more serious disruptions—the proportion of Bacteria rose to 70 ± 7%, while methane-producing microbes became significantly less abundant (Archaea–26 ± 6%, *Methanosarcinaceae*–13 ± 2% *Methanosaeta*–7 ± 2%). The sharp decrease in pH in V5 led to further depopulation of methanogens in the bacterial community, especially for two of the monitored groups—Archaea (20 ± 3%) and *Methanosarcinaceae* (10 ± 3%). Conversely, the taxonomic composition of the LCRF series did not change significantly in response to OLR changes. The share of bacteria ranged from 66 ± 3% in V3 to 68.7 ± 7% in V1. Archaea became progressively more abundant in the anaerobic microbial community (from 27 ± 5% in V1 to 33 ± 5% in V4), whereas *Methanosarcinaceae* rose from 14 ± 3% to 17 ± 4%. Only *Methanosaeta* showed statistically significant variation—from 6 ± 2% to 11 ± 3% of the Archaea community. Other authors have also noted that changing OLRs and pH can induce a disruption in the taxonomic composition of the anaerobic bacterial community [54,64]. Zielińska et al. [54] have investigated taxonomic evolution in an anaerobic reactor used for treating dairy effluent. Changes in process parameters were found to decrease methanogenic bacteria populations, thus impacting the quality and yields of the resultant biogas [54]. Our findings are also corroborated by Boonapatcharoen et al. [64], who noted that an OLR increase from 1.0 to 6.0 kg COD/m^3^·d during anaerobic treatment of cassava starch wastewater caused bacteria to more than double, whereas Archaea to become markedly less abundant [64].

### 3.4. Biogas and Methane Production

**Figure 14:**
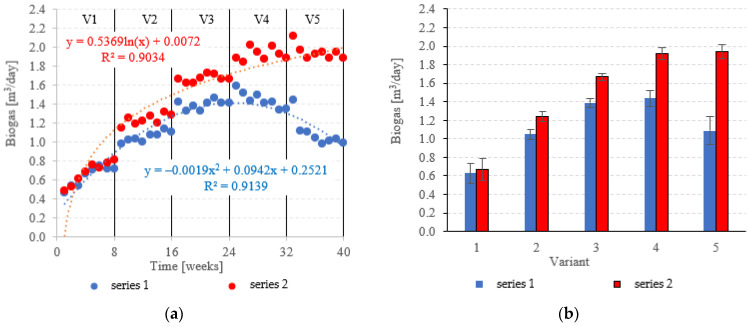
Biogas production ((**a**) changes throughout the experiment; (**b**) average across experimental variants).

**Figure 15:**
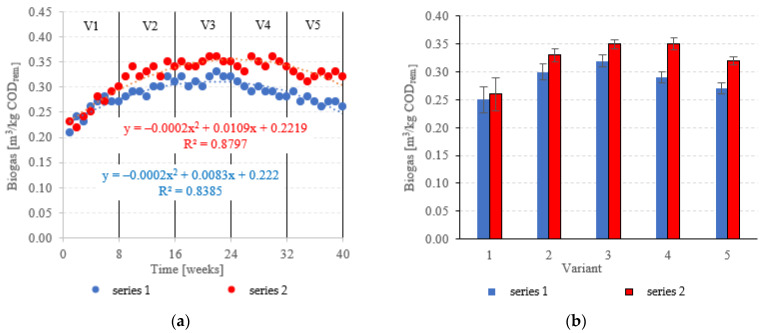
Specific biogas production ((**a**) changes throughout the experiment; (**b**) average specific across experimental variants).

Biogas production in anaerobic reactors is directly affected by the type of filling used, OLR, HRT, conditions in the medium and changes in the microbial populations. Across all of the experimental variants, the LCRF reactors proved superior in terms of digestion performance, with biogas production between 0.67 ± 0.12 m^3^/d in V1 to 1.92 ± 0.06 m^3^/d in V4 (Figure 14a,b). The highest OLR (7.0 kg COD/m^3^·d) did not produce significant further increases in biogas production, peaking at 1.94 ± 0.08 m^3^/d (Figure 14a,b). The LCRF series was also fairly stable in terms of specific biogas production per organic load removed, which ranged from 0.26 ± 0.03 m^3^/kg COD_removed_ in V1 to 0.35 ± 0.01 m^3^/kg COD_removed_ in V4 (Figure 15a,b). The LECAF reactors produced much lower yields of biogas. Specific biogas production peaked at 0.32 ± 0.01 m^3^/kg COD_removed_ in V3 (Figure 15a,b), whereas the highest daily biogas yield of 1.44 ± 0.09 m^3^/d was noted in V4 (Figure 14a,b). The digestion process in series 1 was significantly impaired when the OLR reached 7.0 kg COD/m^3^·d. This variant (V5) yielded 0.27 ± 0.01 m^3^/kg COD_removed_ (Figure 15a,b) and 1.09 ± 0.15 m^3^/d (Figure 14a,b). In a study by Dębowski et al. [33], simulated dairy effluent treated in a magneto-active hybrid anaerobic biofilm reactor yielded a maximum of 0.31 m^3^ biogas/kg COD_removed_ at OLR = 6.0 kg COD/m^3^·d. OLR increases within the range of 5.0 kg COD/m^3^·d to 10 kg COD/m^3^·d were found to impact biogas production. OLRs of 5.0 to 7.0 kg COD/m^3^·d produced biogas yields of 0.30 m^3^/kg COD_removed_. At the 8.0 kg COD/m^3^·d threshold, biogas production in the MA-HAB reactor started to decrease, with the lowest yields (0.12 m^3^/kg COD_removed_) recorded for OLR = 10 kg COD/m^3^·d. Other researchers have also found that active filling had a positive effect on biogas production from model dairy effluent. AF has been demonstrated to improve performance within the COD range of 4.0 kg COD/m^3^ to 6.0 kg COD/m^3^. The effect on biogas production was similar across all AF types [55].

**Figure 16:**
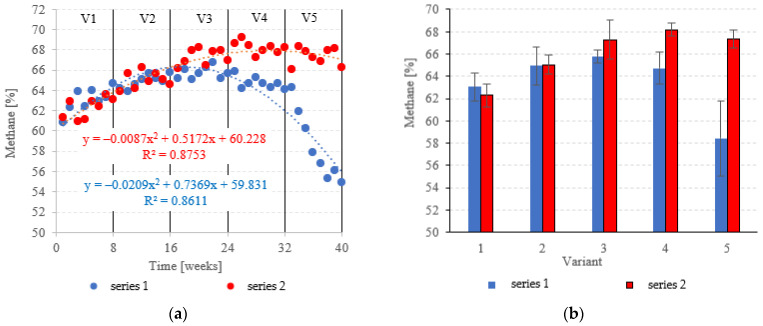
CH_4_ content ((**a**) changes throughout the experiment; (**b**) average across experimental variants).

**Figure 17:**
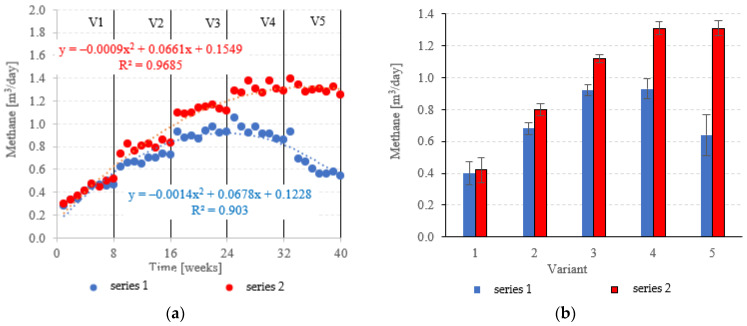
CH_4_ yields ((**a**) daily throughout the experiment; (**b**) average daily across experimental variants).

At OLRs between 3.0 kg COD/m^3^·d and 5.0 kg COD/m^3^·d, CH_4_ in the biogas was similar across both types of fillings. CH_4_ fractions ranged from 63.0 ± 1.2% (V1) to 65.8 ± 0.6% (V3) in series 1 (LECAF), and from 62.3 ± 1.0% (V1) to 67.3 ± 1.7% (V3) in series 2 (LECAF) (Figure 16a,b). However, methane levels started to severely diverge at 6.0 kg COD/m^3^·d OLR and 7.0 kg COD/m^3^·d OLR. Variants 4 and 5 of series 1 produced 64.7 ± 1.4% and 58.4 ± 3.4%, respectively, whereas the same variants of series 2 yielded 68.2 ± 0.6% and 67.3 ± 0.8%, respectively (Figure 16a,b). Biogas production rates and CH_4_ fractions translate directly into daily methane yields. The LCRF series produced higher daily CH_4_ output across all variants, with the sole exception of V1 (adaptation period). The CH_4_ levels ranged from 0.80 ± 0.04 m^3^/d in V2 to 1.31 ± 0.04 m^3^/d in V4 and V5 (Figure 17a,b). By comparison, production in the LECAF reactor ranged from 0.64 ± 0.13 m^3^/d (V5) to 0.93 ± 0.06 m^3^/d (V4) (Figure 17a,b). CH_4_ production efficiency in relation to the COD removed was presented in Table 4.

Other studies on anaerobic treatment of dairy effluent have noted greatly disparate levels of CH_4_ in the biogas—reported fractions range from 25.8 to 83.8% [54]. This variable is driven by multiple factors, including reactor type, process parameters and influent profile [65]. Notably, the filling was used in the top-performing reactors (83.8% CH_4_ in the biogas), as reported by Zielińska et al. [54].

## 4. Conclusions

The present study shows that LCRF ensures high COD removal performance (between 95.2 ± 0.3% and 86.1 ± 2.6%). The LCRF continued to run at steady state even at the highest of the tested OLRs (7.0 kg COD/m^3^·d), whereas the same load induced reactor failure and reduced COD removal (57.2 ± 5.9%) in the LECAF series.

There was little difference in N removal between the two processes. Removal rates ranged between 9.6 ± 1.0% and 12.6 ± 1.3% across all OLRs and both types of filling. On the other hand, P removal was significantly improved by the LCRF (22.1 ± 3.5% to 26.9 ± 4.6%, depending on the OLR). The LECAF anaerobic reactors had little success in removing P_tot._, with removal rates falling within the narrow range of 3.6 ± 1.4% to 5.7 ± 2.2%.

The LCRF reactors proved to be more resistant to OLR increases, with pH holding steady at a near-neutral level and anaerobic bacterial community composition remaining stable across all treatment variants. In the LECAF reactor, on the other hand, the increasing OLRs and decreasing HRTs led to decreased pH and disruptions of the anaerobic bacterial community.

Across all of the experimental variants, the LCRF reactors proved superior in terms of digestion performance, with biogas production ranging from 0.67 ± 0.12 m^3^/d to 1.94 ± 0.08 m^3^/d (depending on the OLR). LCRF reactors also produced more methane in the biogas at the two highest OLRs, with fractions of 68.2 ± 0.6% and 67.3 ± 0.8%.

## Figures and Tables

**Table 1:** Profile of the raw influent fed into the anaerobic reactors.

Parameter	Unit	Mean Value
COD	[mg O_2_/dm^3^]	6020 ± 595
BOD_5_	[mg O_2_/dm^3^]	5317 ± 455
Total P	[mg P/dm^3^]	138.2 ± 35.1
Total N	[mg N/dm^3^]	242.9 ± 51.4
TSS	[mg/dm^3^]	512 ± 94
pH	-	7.75 ± 0.62

**Table 2:** Characteristics of the anaerobic sludge inoculum used in the study.

Parameter	Unit	Value
Water content	(%)	98.8 ± 0.2
Total solids	(g/dm^3^)	29.8 ± 1.7
Mineral solids	(g/dm^3^)	8.6 ± 0.4
Volatile solids	(g/dm^3^)	21.2 ± 0.6
Filtrate COD	(mgO_2_/dm^3^)	630 ± 27.0
Total P in filtrate	(mgTP/dm^3^)	51.0 ± 7.2
Total N in filtrate	(mgTN/dm^3^)	89.2 ± 10.1
pH	-	7.27 ± 0.12

**Table 3:** Composition of the microbial community in the digesters across experimental variants.

Consortium [%]	Series 1				
	V1	V2	V3	V4	V5
Bacteria (EUB338)	69 ± 9	68 ± 7	67 ± 11	70 ± 7	74 ± 8
Archaea (ARC915)	28 ± 5	29 ± 7	29 ± 3	26 ± 6	20 ± 3
*Methanosarcinaceae* (MSMX860)	14 ± 2	15 ± 3	14 ± 2	13 ± 2	10 ± 3
*Methanosaeta* (MX825)	9 ± 2	9 ± 3	9 ± 3	7 ± 2	6 ± 2
**Consortium [%]**	**Series 2**				
	**V1**	**V2**	**V3**	**V4**	**V5**
Bacteria (EUB338)	68 ± 7	67 ± 4	66 ± 3	66 ± 8	68 ± 5
Archaea (ARC915)	27 ± 5	28 ± 2	32 ± 6	33 ± 5	31 ± 3
*Methanosarcinaceae* (MSMX860)	14 ± 3	13 ± 5	17 ± 2	17 ± 4	16 ± 4
*Methanosaeta* (MX825)	6 ± 2	8 ± 3	11 ± 3	11 ± 5	10 ± 2

**Table 4:** CH4 production efficiency in relation to the COD removed.

Series	S1				
Variant	V1	V2	V3	V4	V5
CH_4_ [kg/kg COD_rem._]	0.113 ± 0.017	0.140 ± 0.010	0.151 ± 0.008	0.134 ± 0.007	0.112 ± 0.007
**Series**	**S2**				
**Variant**	**V1**	**V2**	**V3**	**V4**	**V5**
CH_4_ [kg/kg COD_rem._]	0.116 ± 0.021	0.153 ± 0.009	0.168 ± 0.006	0.170 ± 0.008	0.154 ± 0.005

## Data Availability

Not applicable.
